# Alive and well at 100

**DOI:** 10.4056/sigs.1684178

**Published:** 2011-02-25

**Authors:** George M. Garrity, Dawn Field, Nikos Kyrpides

**Affiliations:** 1 Microbiology and Molecular Genetics, Michigan State University, East Lansing, MI; 2 NERC Centre for Ecology and Hydrology, Maclean Building, Wallingford, Oxfordshire, OX10 8BB, United Kingdom; 3 DOE Joint Genome Institute, Walnut Creek, California, USA

A little over eighteen months ago, the Genomic Standards Consortium (GSC), with the support of the Michigan State University Foundation and the U.S. Department of Energy launched Standards in Genomic Sciences (SIGS). It was a bold experiment in open access publishing that was undertaken to fill a set of unmet needs within the broad community of genome biologists and bioinformaticians. It sought to create a new publishing niche with a series of novel article types to provide readers with highly structured and standards-compliant reports on genome and metagenome sequencing projects, in-depth standard operating procedures for analytical pipelines used in genome and metagenome analyses, and in-depth meeting reports, white-papers and other relevant articles to inform the community of ongoing activities of the GSC and other standards formulating bodies within the life sciences.

With the publication of this issue of SIGS, we are pleased to announce that we have passed two important milestones. The first, which is of significance to authors, is that articles appearing in SIGS are now archived in PubMed Central and all of our content is available through the PubMed Central site, as well as the redesigned SIGS site. The second milestone is that over 100 short genome reports have now been published in SIGS. Based on the number of published genome reports, this places SIGS in third place among all journals publishing genome reports. Equally impressive is the acceptance of SIGS by the community at large. Articles appearing in SIGS have been downloaded over 50,000 times, and that rate has seen a steady increase over time.

Of course, the success of any scientific publication is a function of the quality of the articles that it publishes. This can only happen when there are dedicated authors and reviewers. This is especially true for a start-up publication that does not have an established publication trail. Sustainability is also a concern for any organization publishing an open access journal. We were quite fortunate to have our start-up costs covered by two grants. However, those grants will soon come to an end and we will need to charge a modest publication fee to cover production costs. We are indebted to those authors who took a chance and published their findings in SIGS and to Michigan State University and the Department of Energy for their support. We look forward to SIGS becoming the publication of choice for genome and metagenome articles, SOPs, meeting reports and other articles of interest to the community in the future.

